# Oncoplastic breast surgery: initial experience at the Centro Clinico de Estereotaxia—CECLINES, Caracas, Venezuela

**DOI:** 10.3332/ecancer.2014.470

**Published:** 2014-10-14

**Authors:** V Acosta-Marin, V Acosta-Freites, A Contreras, R Ravelo, G Fuenmayor, C Marin, A Ramirez, M Acosta-Marin, J Perez-Fuentes, I Longobardi, H Esteves

**Affiliations:** 1 Breast Surgery Department, Centro Clinico de Estereotaxia—CECLINES, Caracas, Venezuela; 2 Breast Pathology Department, Centro Clinico de Estereotaxia—CECLINES, Caracas, Venezuela; 3 Breast Imaging Department, Centro Clinico de Estereotaxia—CECLINES, Caracas, Venezuela; 4 Breast Nurse Department, Centro Clinico de Estereotaxia—CECLINES, Caracas, Venezuela

**Keywords:** breast cancer, conservative surgery, oncoplastic surgery, Venezuela, satisfaction, mammoplasty

## Abstract

**Background:**

Breast-conserving surgery (BCS) may sometimes lead to deformities in the remaining breast. Oncoplastic surgery (OPS) aims to improve our aesthetic results even in the case of major volume resections. The purpose of this study is to provide an objective evaluation of our initial experience with OPS, mainly based on the levels of satisfaction reported by both patients and surgeons.

**Patients and Methods:**

This prospective study was performed at CECLINES in Caracas, Venezuela, between January 2011 and October 2012. It involved 107 consecutive patients in two groups: 52 patients with level II OPS versus 55 patients with ‘standard’ BCS (SBCS). We evaluated the level of satisfaction and cosmetic outcome at 6 and 12 months post-operation using a score from 1 (bad) to 5 (excellent). The cosmetic score was recorded during the follow-up by the surgeon, by phone calls, and photographs were reviewed by a panel of four observers.

**Results:**

The participation rate in the cosmetic outcome/level of satisfaction evaluation was 100% at 6 months and 96.2% at 12 months. The average tumour size was 23 mm [standard deviation (SD): 13.5] for the OPS group versus 17.6 mm (SD: 8.3) for the SBCS group (*p* = 0.017). The average weight for the surgical specimen was 101 g (range: 30–512 g) in the OPS group versus 60.4 g (range: 20–135 g) in the SBCS group (*p* = 0.004). The OPS techniques most performed were round block 40.3% (21/52), inverted T mammoplasty 26.8% (14/52) and vertical scar mammoplasty 15.3% (8/52). Of all the patients, 51.9% (27/52) had symmetrisation procedures performed distributed according to the period of the study: 77.2% (17/22) in 2011, 56.6% (17/30) in 2012, and 18.1% (6/33) in 2013. The rate of complications was 5.7% (3/52) in the OPS group and 0% for the SBCS group (*p* < 0.005). The average cosmetic score at 6 months by patients in the OPS group was 4.4; patient satisfaction scores of 4 (good) and 5 (excellent) were 88.4%. In the SBCS group at 6 months, the mean score reported by patients was 4.2, with scores 4–5 being 83.4% (*p* = 0.644). The cosmetic score by surgeons in the OPS group at 6 months was 4.5; the surgeon satisfaction scores of 4–5 were 94.2%. In the SBCS group, the surgeons’ mean score at 6 months was 4.1, with 84.5% of scores being 4 or 5 (p < 0.005). The final cosmetic score by patients in the OPS group at 12 months was 4.5; patient satisfaction scores of 4–5 were 90.4%. In the SBCS group, the final mean score at 12 months by patients was 4.2, with 77.5% of scores being 4 or 5 (p < 0.005). The final cosmetic score by surgeons in the OPS group at 12 months was 4.5; surgeon satisfaction scores of 4–5 were 92.3%. In the SBCS group, the surgeons’ final mean score at 12 months was 4.1, with 84.5% of scores being 4 or 5 (p < 0.005).

**Conclusions:**

OPS provides good satisfaction rates. An SBCS when an OPS is not indicated mostly results in good satisfaction levels and cosmetic scores. Usually, the results remain stable after 6 months. The use of OPS allows the excision of bigger lesions and surgical specimens. Symmetrisation procedures are not always required. With the appropriate patient selection, the rate of complications is low for both OPS and SBCS.

## Background

For a long time now, breast-conserving surgery (BCS) has proven to be as good as mastectomy for the surgical treatment of early breast cancer [1]. Results from randomized trials have proven the oncological safety of breast-conserving therapy (BCT) [2]. Despite these excellent results, this surgical approach may sometimes lead to deformities in the remaining breast. Since 1994, we have had a new way to approach BCS: oncoplastic surgery (OPS) [3]. In 2010, Clough *et al* published a classification of different procedures in order to simplify our decision-making process and to improve our aesthetic results even in major volume resections [4]. This approach not only allows wide excisions with adequate margins but it also has very good reported recurrence rates [5, 6]. Another interesting classification is the one developed by Benigno Acea from Complexo Hospitalario Universitario A Coruña in Spain, ‘the segmentation theory’, in which the breast is divided the breast in eight segments, depending mainly on the breast size and ptosis, which helps to predict the consequences of local resection in each breast segment and, at the same time, optimise the choice of the best procedure to prevent deformities [7].

The level of satisfaction for OPS has been studied in several publications, reaching very optimistic rates by both doctors and patients [5, 8, 9]. Recently, Veiga *et al* reported high rates of satisfaction in patients on whom OPS was performed [9]. There are no reports of this in Venezuela until now. The aim of this study is to give an objective evaluation of our initial experience with oncoplastic techniques for breast surgery compared to those patients in whom a ‘traditional’ BCS was performed, mainly based on the levels of satisfaction by both patients and surgeons. We will also evaluate some demographic, clinical, and surgical data that resulted from the evaluation and treatment of these patients.

## Methods

This prospective study was performed at the Centro Clinico de Estereotaxia (CECLINES) in Caracas, Venezuela, during the period from January 2011 to October 2012. It involved 107 consecutive patients in two groups: 52 patients with level II OPS versus 55 patients with ‘standard’ BCS (SBCS). We excluded all patients who had a mastectomy, had a previous breast surgery due to breast cancer, did not have enough information, or did not reach at least 12 months of follow up.

**Figure 1.**
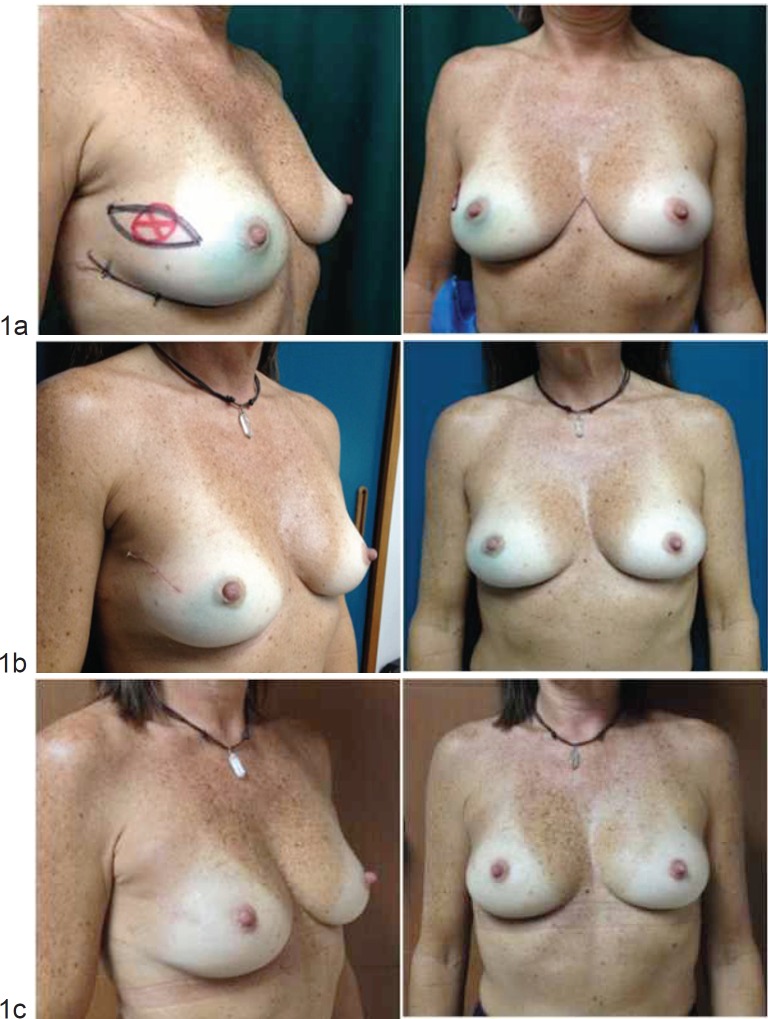
Standard BCS: (a) before the operation, (b) 6 months after the operation, and (c) 12 months after the operation.

**Figure 2.**
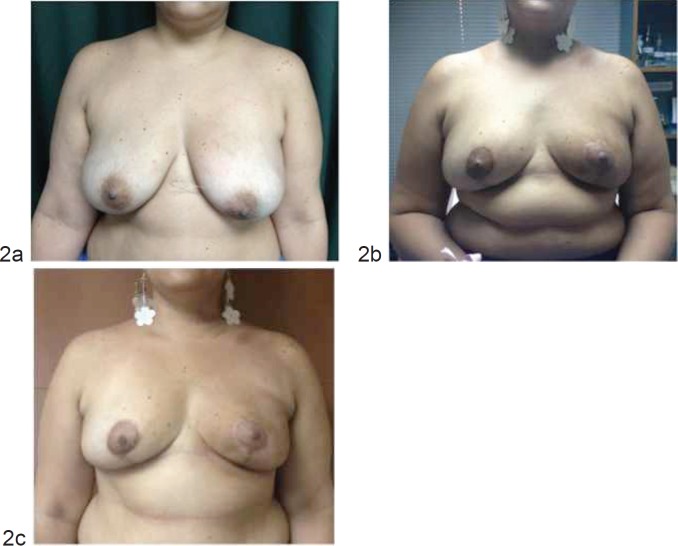
Oncoplastic breast surgery: (a) before the operation, (b) 6 months after the operation, and (c) 12 months after the operation.

The study’s main purpose was to evaluate the cosmetic outcome and level of satisfaction between the two surgical techniques (OPS versus SBCS) by analysing the appreciation of both patients and surgeons at 6 and 12 months post operation (Figures 1 and 2). The scores for the cosmetic outcome and the level of satisfaction were recorded in a punctuation described in previous experiences being 1 = bad, 2 = poor, 3 = fair, 4 = good, and 5 = excellent [5, 6, 10, 11]. The cosmetic outcome was recorded during the follow up by the surgeon, by phone calls, and photographs were reviewed by a panel of four observers: one breast surgeon, two surgical oncologists, and one plastic surgeon. Pictures were taken with the patient shown in front-facing position, at +45° and -45°. We consider a score of 4 (good) and 5 (excellent) as a measure of satisfaction. We also recorded important variables, such as the oncoplastic technique used, breast cup size (A,B,C,D), body mass index (BMI), tumour size, surgical specimen’s weight and complications.

All the operations were performed by the same surgical team: two breast surgeons and one surgical oncologist.

SBCS is defined as a partial resection of the breast followed by post-operative radiotherapy. OPS refers to several surgical techniques by which segments of breast tissue are removed to achieve wide margins around the tumour while the remaining glandular tissue is transposed to achieve the best possible aesthetic outcome [9, 12, 13]. Level II OPS was defined as a procedure which requires major volume resection and encompasses a mammoplasty technique [4]. These ‘therapeutic mammoplasties’ involve extensive skin excision and breast reshaping [14].

### Statistical analysis

We calculated the differences in nominal variables between groups using the chi-square test; in the case of continuous variables, we used the Student’s *t* test. We considered statistical significance level of *p* < 0.05, and we used JMP-SAS implementation version 12.

## Results

The participation rate of patients in the cosmetic outcome and the level of satisfaction were 100% at 6 months and 96.2% at 12 months (52 in the OPS group and 51 in the SBCS group). The average age for the OPS group was 54.2 years (SD 10.6) and 58.1 years (SD 12.1) for the SBCS group (*p* = 0.081). The average tumour size was 23 mm [standard deviation (SD): 13.5] for the OPS group and 17.6 mm (SD: 8.3) for the SBCS group (*p* = 0.017). The breast cup sizes were A–B 72.3% for the OPS group and 91.3% for SBCS group, C–D 27.7% for the OPS group and 8.7% for the SBCS group (*p* = 0.018). The average weight for the surgical specimen was 101 g (30–512) for the OPS group and 60.40 g (20–135) for the SBCS group (*p* = 0.004). The patients’ BMI was 27.2 kg/m^2^ (SD: 4.6) for the OPS group and 26.1 kg/m^2^ (SD: 5.2) for the SBCS group (*p* = 0.892) (Tables 1 and 2).

**Figure 3.**
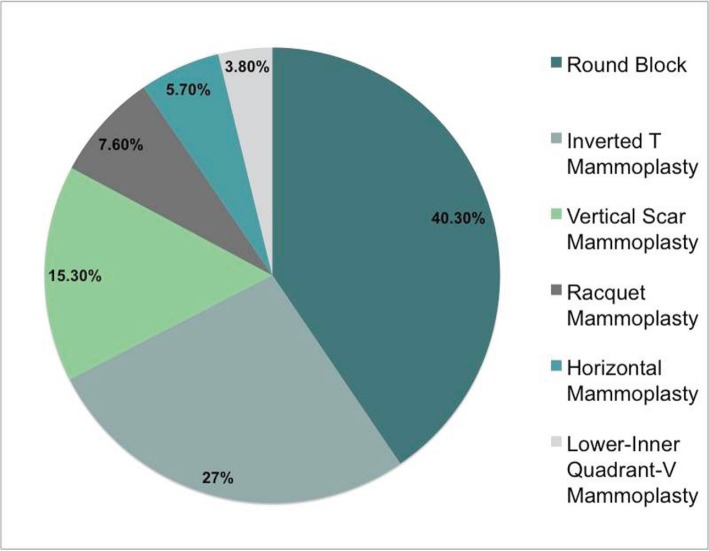
OPS techniques performed.

The OPS techniques performed were round block 40.3% (21/52), inverted T mammoplasty 26.8% (14/52), vertical scar mammoplasty 15.3% (8/52), racquet mammoplasty 7.6% (4/52), horizontal mammoplasty 5.7% (3/52), and LIQ V mammoplasty [4, 7, 11] 3.8% (2/52) (Figure 3). 51.9% (27/52) of patients had symmetrization procedures performed, distributed according to the period of the study as follows: 77.2% (17/22) in 2011, 56.6% (17/30) in 2012. The rate of complications was 5.7% (3/52) in the OPS group (fat necrosis, infection, and areola slough) and 0% for the SBCS group (*p* < 0.005) (Table 3).

**Figure 4.**
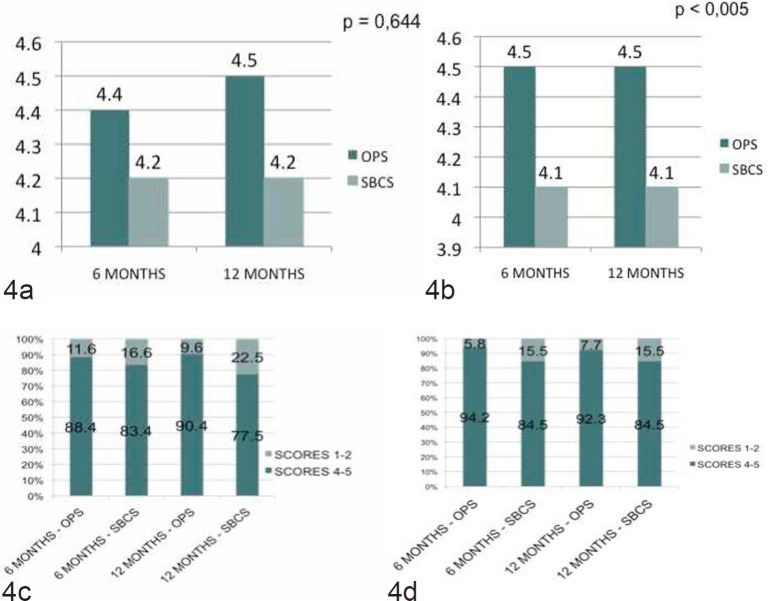
(a) The cosmetic score by patients at 6 and 12 months, (b) the cosmetic score by surgeons at 6 and 12 months, (c) the satisfaction score by patients at 6 and 12 months, and (d) the satisfaction score by surgeons at 6 and 12 months.

The average cosmetic score at 6 months by patients in the OPS group was 4.4; patient’s satisfaction scores of 4 (good) and 5 (excellent) were 88.4%. In the SBCS group at 6 months, the mean cosmetic score by patients was 4.2; patient satisfaction scores of 4 (good) and 5 (excellent) were 83.4% (*p* = 0.644) (Figure 4a–c). The average cosmetic score by surgeons in the OPS group at 6 months was 4.5; surgeon satisfaction scores of 4 and 5 were 94.2% (Figure 4b–d). In the SBCS group, the surgeons’ mean cosmetic score at 6 months was 4.1; surgeon satisfaction scores of 4 and 5 were 84.5% (*p* < 0.005) (Figure 4b–d).

The final average cosmetic score by patients in the OPS group at 12 months was 4.5; patient’s satisfaction scores of 4 (good) and 5 (excellent) were 90.4% (Figure 4a–c). In the SBCS group, the patients’ final mean cosmetic score at 12 months was 4.2; patient satisfaction scores of 4 (good) and 5 (excellent) were 77.5% (*p* < 0,005) (Figure 4a–c). The final average cosmetic score by surgeons in the OPS group at 12 months was 4.5; surgeon satisfaction scores of 4 and 5 were 92.3%. In the SBCS group, the surgeons’ final mean cosmetic score was 4.1; surgeon satisfaction scores of 4 and 5 were 84.5% (*p* < 0.005). (Figure 4b–d).

The evaluation at 12 months did not show any significance between BMI and appreciation by patients or surgeons in either group. Patient’s appreciation at 12 months according to BMI in the OPS group was *p* = 0.172 versus *p* = 0.425 in the SBCS group, and surgeon’s appreciation at 12 months according to BMI in the OPS group was *p* = 0.222 versus *p* = 0.176 in the SBCS group (Tables 4 and 5).

## Discussion

The evolution of breast surgery has allowed us not only to be more strict in the oncological and safety approach to our patients but also to considerably improve cosmetic results, as there are no classifications that can lead us to choose the best technique according to the localization, size, breast density and volume to be excise in order to prevent deformities [4, 7, 12, 15].

It is extremely important in any breast centre to improve the quality of breast cancer care. The evaluation of satisfaction, after surgical approaches in both ‘traditional’ breast surgery and new techniques, such as OPS, is a way to avoid estimations and subjectivity and also to develop strategies that improve our current practice.

In our study, there was no statistical difference in age between the two groups.

The average tumour size to be excised was bigger in the OPS group compared to the SBCS group, 23 mm versus 17.6 mm (*p* = 0.017). This corresponds to recent publications, such as the meta-analysis by Losken *et al* where the tumour size for the OPS group was bigger than for the BCT group, 2.7 cm versus 1.2 cm. Our study demonstrates a surgical specimen weight of 101 g for the OPS group and 60.4 g for the SBCS group (*p* = 0.004). In 2003, Clough *et al* published an average of excised tissue from the breast, with the lesion weight being 222 g (range 20–1450) [6]. Kaur *et al* performed a prospective trial comparing quadrantectomy alone (*n* = 30) and resection with oncoplastic reconstruction (*n* = 30), demonstrating larger resection weights in the oncoplastic reconstruction group (200 versus 118 g) (*p* = 0.16) [16].

The tumour size in relation to breast size is one of the most important factors when attempting to obtain a cosmetically favourable result. A conflict exists, therefore, between performing a resection wide enough to obtain optimal oncologic control and not removing so much breast tissue as to leave a deformity or a large discrepancy compared with the other side [6]. Even though there was no statistical difference between the groups in our study according to the cup size, both groups were mainly cup A–B (OPS group: 72.34%, and SBCS group: 91.3%), which explains to some extent why our surgical weight is lighter than in other publications.

Some authors have described a higher BMI as a risk factor for poor aesthetic outcome [17]. In our study, the BMI for the OPS group was 27.2 kg/m^2^ (SD 4.6) and 26.1 kg/m^2^ (SD 5.2) for the SBCS group (*p* = 0.892). The analysis between BMI and patient’s surgeon’s appreciation at 12 months did not show any correlation between BMI and poor outcome.

In our initial experience, the most common OPS technique used was the round block (40.3%), followed by the inverted T Mammoplasty (26.8%). Whenever the breast–tumour size ratio, the breast density, and the tumour localization allow a round block technique, this is the optimal way to approach patients with small breasts, and that might be an explanation why we have such a high percentage of this technique. We mostly use this technique for lesions located near the areola and in the upper inner quadrant (UIQ), but when the right patient is selected, it can give you access to almost any quadrant of the breast [18]. One important characteristic that must be taken into account for possible candidates for this technique is the breast density, as it requires a broad undermining, and in a fatty breast this would not be possible. The average of inverted T mammoplasty in our study (26.8%) corresponds to other recent publications, such as the paper by Clough *et al* in his report of OPS at The Paris Breast Centre on 175 patients in which 42 patients (24%) had an inverted T mammoplasty performed [5].

There is some evidence that patients’ satisfaction is correlated with post-operative complications and breast asymmetry [19]. In our study, 51.9% of patients had a procedure to correct an asymmetry. The rate of symmetrisation procedures during our oncoplastic practice has evolved from 77.2% (17/22) in 2011 to 56.6% (17/30) in 2012 to 18.1% (6/33) in 2013 (2013 data not on the paper analysis). This last due to the fact that during the evolution of our oncoplastic practice we have begun to notice that what other authors have pointed out as asymmetry is well accepted when it is not severe [19], while not performing a contralateral procedure might result in less probability of complications and less probability of delayed adjuvant treatment with good satisfaction rates. Regarding this issue, Clough *et al* recently reported only 26.9% procedures for breast symmetry rating 85% of satisfaction scores good and very good [5].

There are reports of complications after OPS that range from 1.7% to 20% [5, 8, 20, 21]. These complications may result in delayed adjuvant treatment [22]. In our study, the complication rate in the OPS group was 5.7%; no complication was observed in the SBCS group (*p* < 0.005). This did not lead to any delay in the initiation of adjuvant treatment as the complications were an infection, a fat necrosis, and an areola slough. One reason to explain the low rate of complication is our strict selection of patients for OPS techniques. Clough *et al* demonstrated that the inclusion of breast density as a third key element in their decision-making process reduced the delayed wound healing from 9% to 5% [5].

The level of satisfaction in OPS surgery has been published by some groups [5, 9, 20, 23]. This has not been reported by any surgical team in Venezuela until now. Recently, Veiga *et al* in Brazil performed a trial that assessed differences due to surgeons’ gender and surgical specialty in the evaluation of aesthetic outcomes of oncoplastic surgery as well as patients’ appreciation. In that study, compared to the control group, patients and all the raters attributed higher grades to result in the oncoplastic group [9]. This corresponds to our results as the patient average scored at 12 months follow-up was 4.5 in the OPS group against 4.2 in the SBCS group, and surgeons’ appreciation at 12 months had a score of 4.5 in the OPS group against 4.1 in the SBCS group. Losken *et al* also published that when aesthetic and patient satisfaction was evaluated in their meta-analysis, they found that the overall satisfaction in the BCT group alone was 80%, compared to 90% in the oncoplastic group [20]. According to our findings, patients satisfaction in the OPS group at 12 months was 90.4% and in the SBCS 77.5%. L’Institut du Sein in Paris also reported in 2012 the mean cosmetic score in 80 patients with OPS techniques in 4.6 (scale: 1 = poor – 5 = excellent) and the cosmetic scores were 4 and 5 in 68 patients (85%) [5]. In our study, at 12 months surgeons appreciation in the OPS group was 92.3% and for the SBCS 84.5%. Compared to SBCS group, patients and surgeons attributed higher grades to results of OPS group through all the study (6 and 12 months). Some authors have stated that in oncoplastic patients aesthetic results significantly increased over time. In our study, the results demonstrate that cosmetic score remains stable from 6 months onwards, as for surgeons the cosmetic score for the OPS group is 4.5 for both 6- and 12-month post-operative evaluations and 4.1 for the SBCS in both periods (Figure 4b). The same happened for patient’s appreciation, with the scores being 4.4 in the 6-month evaluation and 4.5 in the 12-month evaluation for the OPS group, and 4.2 for both 6 and 12-month post-operation in the SBCS group (Figure 4a).

There are some limitations to our study. The number of patients involved is low; nevertheless, the lack of a bigger group of study and being in a centre devoted to breast cancer care allowed a high level of adherence to the study, 96.2% at 12 months. We do recognize that another limitation is the small number of raters and the need for a more accurate and objective method of satisfaction measurement in order to avoid any bias.

## Conclusion

The OPS technique definitely provides good satisfaction rates from both patients and surgeons. A SBCS when an OPS technique is not indicated mostly returns good satisfaction levels and cosmetic scores. Usually, the results remain stable after 6 months. The use of OPS techniques allows the excision of bigger lesions and heavier surgical specimens and is more applicable to patients with a larger brassiere cup. Symmetrization procedures are not always required. With the appropriate patient selection, the rate of complications is very low for both OPS and SBCS groups.

## Figures and Tables

**Table 1.** Demographic and surgical variables.

	Level II OPS group *n* = 52 Average	SBCS group *n* = 55 Average	
Age (SD)	54.2 y.o (10.6)	58.1 y.o (12.1)	*P* = 0.081
BMI (SD)	27.2 kg/m^2^ (4.6)	26.1 kg/m^2^ (5.2)	*P* = 0.892
Tumour size (SD)	23 mm (13.5)	17.6 mm (8.3)	*P* = 0.017
Surgical specimen weight (Range)	101 g (30–512)	60.4 g (20–135)	*P* = 0.004

SD: Standard Deviation; y.o: years old

**Table 2.** Pre-operation breast cup size.

	Level II OPS group *n* = 52	SBCS group *n* = 55	
A–B (%)	72.3	91.3	
C–D (%)	27.7	8.7	
Total	100	100	
			*P* = 0.018

**Table 3.** Complications.

	Level II OPS group *n* = 52	SBCS group *n* = 55	
Fat Necrosis	1	0	
Infection	1	0	
Areola Slough	1	0	
Total	3 (5.7%)	0	
			*P* < 0.005

**Table 4.** The relationship between patients’ appreciation and BMI according to each group, OPS and SBCS.

Group			Patient’s appreciation at 12 months			Total	
			Poor/Bad	Fair	Good/Excellent		
OPS	BMI	Normal	0	0	18	18	
		Overweight	1	3	16	20	
		Obese	1	0	13	14	
	Total		2	3	47	52	*P* = 0.172
SBCS	BMI	Normal	0	3	15	18	
		Overweight	0	4	15	19	
		Obese	1	3	10	14	
	Total		1	10	40	51	*P* = 0.425

**Table 5.** The relationship between surgeons’ appreciation and BMI according to each group, OPS and SBCS.

Group			Patient’s appreciation at 12 months		Total	
			Fair	Good/Excellent		
OPS	BMI	Normal	0	18	18	
		Overweight	3	17	20	
		Obese	1	13	14	
	Total		4	48	52	*P* = 0.222
SBCS	BMI	Normal	3	16	19	
		Overweight	2	16	18	
		Obese	3	11	14	
	Total		8	43	51	*P* = 0.176
